# An improved method for the segmentation of roots from X-ray computed tomography 3D images: Rootine v.2

**DOI:** 10.1186/s13007-021-00735-4

**Published:** 2021-04-08

**Authors:** Maxime Phalempin, Eva Lippold, Doris Vetterlein, Steffen Schlüter

**Affiliations:** 1 Department of Soil System Science, Helmholtz Centre for Environmental Research GmbH-UFZ, Halle, Germany; 2 Martin-Luther-University Halle-Wittenberg, Institute of Agricultural and Nutritional Sciences, Halle, Germany

**Keywords:** High-throughput root phenotyping, Image analysis, Root segmentation, Root system architecture, Cylindrical feature detection, X-ray computed tomography, Root diameter

## Abstract

**Background:**

X-ray computed tomography is acknowledged as a powerful tool for the study of root system architecture of plants growing in soil. In this paper, we improved the original root segmentation algorithm “Rootine” and present its succeeding version “Rootine v.2”. In addition to gray value information, Rootine algorithms are based on shape detection of cylindrical roots. Both algorithms are macros for the ImageJ software and are made freely available to the public. New features in Rootine v.2 are (i) a pot wall detection and removal step to avoid segmentation artefacts for roots growing along the pot wall, (ii) a calculation of the root average gray value based on a histogram analysis, (iii) an automatic calculation of thresholds for hysteresis thresholding of the tubeness image to reduce the number of parameters and (iv) a false negatives recovery based on shape criteria to increase root recovery. We compare the segmentation results of Rootine v.1 and Rootine v.2 with the results of root washing and subsequent analysis with WinRhizo. We use a benchmark dataset of maize roots (*Zea mays* L. cv. B73) grown in repacked soil for two scenarios with differing soil heterogeneity and image quality.

**Results:**

We demonstrate that Rootine v.2 outperforms its preceding version in terms of root recovery and enables to match better the root diameter distribution data obtained with root washing. Despite a longer processing time, Rootine v.2 comprises less user-defined parameters and shows an overall greater usability.

**Conclusion:**

The proposed method facilitates higher root detection accuracy than its predecessor and has the potential for improving high-throughput root phenotyping procedures based on X-ray computed tomography data analysis.

## Introduction

X-ray computed tomography (CT) is acknowledged as a powerful tool for the study of root system architecture of plants growing in soil. However, the study of the root system architecture is only possible after performing root segmentation, i.e., the binarization of the grayscale data into root voxels and background voxels. Root segmentation is often regarded as a tedious and difficult task as its success depends on several factors such as the image resolution, the signal-to-noise ratio during image acquisition and the gray value (GV) contrast between the roots and all other surrounding features in soil [1].

In the past years, many methods have been developed to segment and visualize roots in tomograms acquired with X-ray CT [1–17]. Some algorithms rely on simple thresholding methods [8]. With these methods, the roots are segmented based on a histogram analysis and a GV criterion. These methods usually fail at segmenting roots properly because of the overlapping GV of roots, water, organic matter and the soil matrix. The GV at the edges of roots, water, organic matter and soil matrix also show gradual changes of intensity spanning several voxels rather than a crisp intensity step [18]. This effect, known as the “partial volume effect”, is also responsible for poor segmentation results when using simple thresholding methods. More advanced thresholding methods rely on the use of adaptive local thresholding values (also referred to as “Region growing”) which use an additional connectivity criterion to binarize the root and background voxels [3]. For both the simple and the adaptive thresholding methods, there is an inherent trade-off to be made by the user. If the GV range assigned to roots bounded by two thresholds is too broad, over-segmentation may occur (i.e., segmented root edges extend into the surrounding features and appear frayed) and the false positives need to be removed through user-interaction which is a subjective, tedious and time-consuming task. Inversely, if the GV range is too narrow, an important loss of roots may occur which biases the root system architecture analysis of the scanned sample. To tackle the issue of overlapping GV of roots and other materials, root tracking methods such as the “RooTrak” algorithm have been developed [10]. With this method, the volumetric data is viewed as a sequence of X–Y cross-sectional images aligned along the Z axis. As the 3D stack is explored, root cross sections appear to move in the image and such “movements” can be used to reconstruct the root system. Methods relying on deep-learning algorithms and multi-scaled based approaches have also become common in X-ray CT and magnetic resonance imaging. promising applications of deep learning for the segmentation of roots from X-ray CT data were recently demonstrated by [13].

Gao et al. [6] proposed a new algorithm to segment root systems growing in soil by exploiting a typical morphological characteristic of the roots, i.e., their cylindrical shape. This approach was first introduced for vessel detection in medical imaging [19]. The vessel enhancement filter was later adopted to segment roots in 3D Magnetic Resonance Imaging data [20]. The rationale of this method is that the cylindrical shape of roots is unique among all materials and features found in soil. The shape-based semi-automated algorithm is named “Rootine” [6] and has shown to outperform the “Root1” [4] and “Region growing” [3] methods in terms of root recovery and segmentation accuracy. This demonstrated promising future applications of the algorithm for high-throughput root phenotyping based on X-ray CT data analysis. However, the Rootine algorithm relies on a substantial number of parameters to be calibrated by the user. Moreover, Rootine suffers from the fact that some of the parameters and their effects are difficult to identify and to interpret by a non-experienced user.

In this paper, we aim at developing an improved method for the segmentation of roots from 3D X-ray CT images that overcomes the aforementioned drawbacks of Rootine. The objectives of this work are then to develop a new Rootine version (i.e., “Rootine v.2”) for which the segmentation accuracy and the user friendliness are increased. Specific objectives are to propose a second version in which (i) the root recovery is higher, (ii) the segmented root diameters are better captured, (iii) segmentation artefacts are reduced, (iv) the number of tunable parameters is reduced and (v) the parameters are related to root properties (i.e., their GV, shape and connectivity).

The ability of the new segmentation algorithm to fulfill these criteria is evaluated by systematic comparison with the former algorithm Rootine (which will be referred to as “Rootine v.1”) and the results obtained by conventional, destructive root sampling and analysis of the washed-off roots with the software WinRHIZO. In addition, a comparison between both algorithms is made by considering aspects such as the runtime and the overall usability of the algorithms. In that respect, the benchmark dataset of the “worse case” scenario presented in [6] is used. This benchmark dataset was chosen to test Rootine v.2 as it presents several challenges to overcome, namely the presence of high soil heterogeneity, a poor quality of the images (i.e., a low number of projections during image acquisition) and a rather low image resolution as compared to the diameter of the roots to segment. The two first challenges contributed to a deterioration of the signal-to-noise ratio whereas the third exacerbates the partial volume effect at the edges of the fine roots. In the study of [6], these challenges led to a rather low root recovery of (i.e., 29%) in comparison with conventional root sampling. These challenges combined make the benchmark dataset of [6] a perfect candidate for further testing and improvements of root segmentation algorithms.

In order to show that the improvements in segmentation quality are not solely due to an overfitting of Rootine v.2 for this particular dataset, we also demonstrate the performance of the new version on the so called “best case” scenario dataset of [6]. In this “best case” scenario, the soil and the scan settings were chosen in order to create low soil heterogeneity and a high signal-to-noise ratio. Those two aspects contributed to a robust estimation of root length (i.e., 99% of recovery) in comparison with root washing and analysis with WinRHIZO. Finally, a 3D visual comparison of the results obtained with Rootine v.2, Rootine v.1, Root1 and the region growing method available in VG Studio Max 2.1 is provided for a small test cube image of the worse case scenario.

## Material and methods

### Plant growth conditions and destructive sampling

Maize plants (*Zea mays* L. cv. B73) were grown in repacked soil sieved down to ≤ 2 mm particle size. The plants were grown in a climate chamber for 21 days in cylindrical containers of 7 cm inner diameter and 23 cm height. Six plants were analyzed for each scenario. One day after X-ray CT scanning, the plants were harvested and the pots were cut in several layers of 4 cm. The roots in those layers were washed off with deionised water and stored in a 50% ethanol solution prior to analysis. In order to assess root length density (RLD) for each layer, root samples were scanned with a flatbed scanner (EPSON perfection V700) and the obtained images were analyzed with WinRHIZO Pro™ (Version 2019a, Regent Instruments, Canada). In total, twelve layers were investigated for each scenario (i.e., two per growing pot, one at the top and one at the bottom). For detailed information on the plant growth conditions and the destructive root sampling method, the reader is referred to [6].

### Workflow of Rootine v.2

**Fig. 1:**
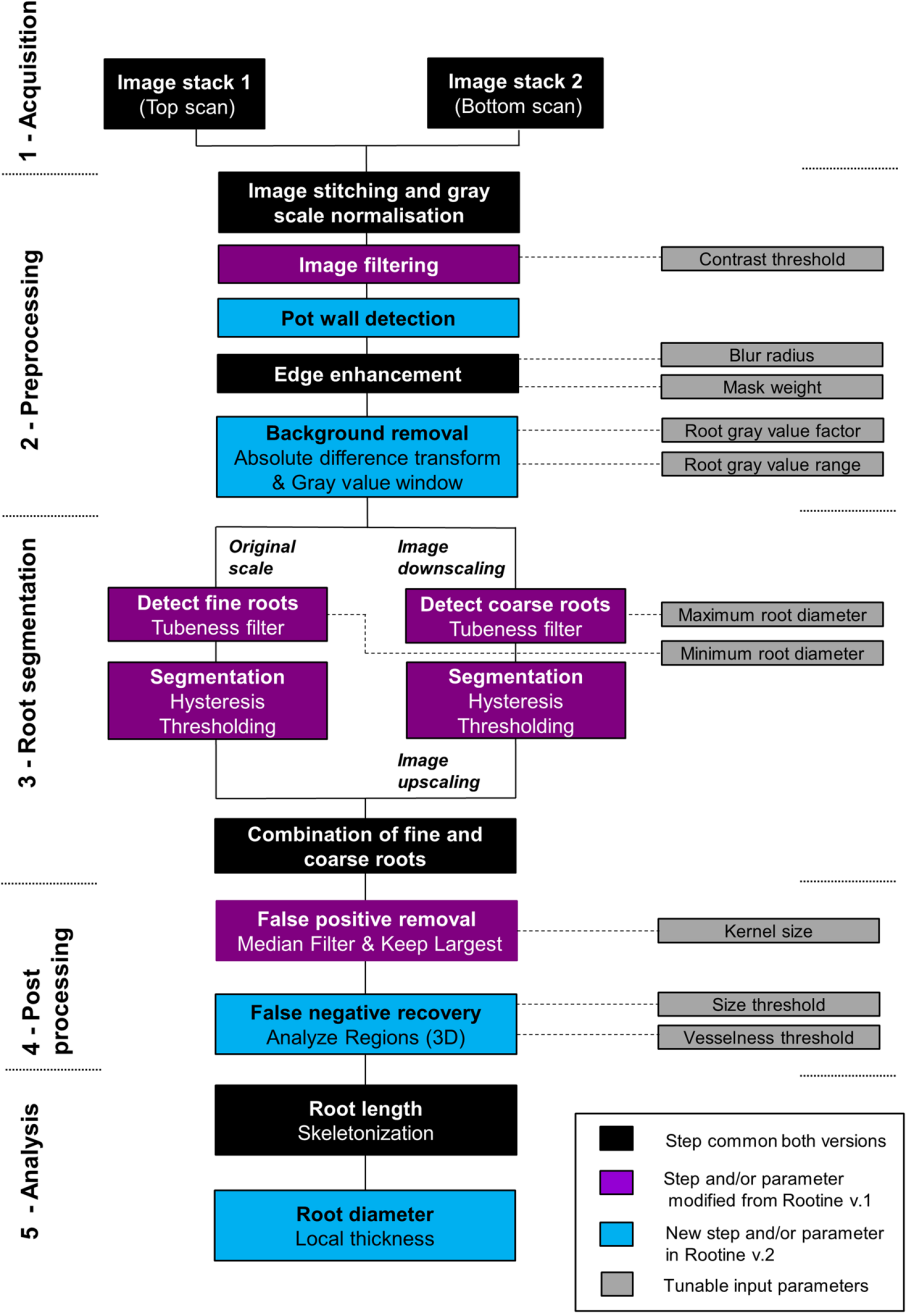
Synoptic view of the Rootine v.2 workflow including the comparison with Rootine v.1

The workflow of Rootine v.2 is synoptically shown in Fig. 1 where the novelties of the algorithm are highlighted in blue and the steps and/or parameters that were modified from the original Rootine v.1 are shown in purple.

#### Image acquisition

3D X-ray CT images were acquired and reconstructed into an 8-bit grayscale 3D tomogram having a voxel size of 45 μm. The 8-bit conversion allows saving space without considerable loss of information. During the 8-bit conversion, contrast was optimized using a percentile stretching method, i.e., 0.2% of the darkest and brightest voxels are set to 0 and 255, respectively. A linear stretching is applied for all GV between 0 and 255. Considering the geometry of the panel detector of the X-ray CT device (X-TEK XTH 225, Nikon Metrology), pots were scanned at two depth intervals (i.e., a bottom and top depth) making sure that an overlapping region was present. For more information regarding the image acquisition procedure, the reader is here again referred to [6].

#### Preprocessing

Before concatenation of the bottom and the top images, the overlapping regions in both scans are removed using the “Slice remover” function available in the free software ImageJ [21]. After concatenation, the obtained image comprises 3000 voxels in the Z dimension, 1750 voxels in the X and Y dimension and has a size ≈ 8.6 GB. The vertical extent of the stack is 13.5 cm. At the boundary of the two stitched images in the concatenated stack, a GV discontinuity is present due to an illumination drift caused by the X-ray CT hardware (see Fig. S2 in [6]). This GV discontinuity is corrected for using the “Attenuation correction” plugin [22] in ImageJ. This correction applies a linear transformation of GV to each slice of the stack in order to make the average and standard deviation of the background constant and equal to that of a reference slice throughout the stack [22]. Note that this GV discontinuity is specific to the X-ray CT hardware used in the benchmark dataset of [6] and that this step might not be necessary with other set-ups.

**Fig. 2:**
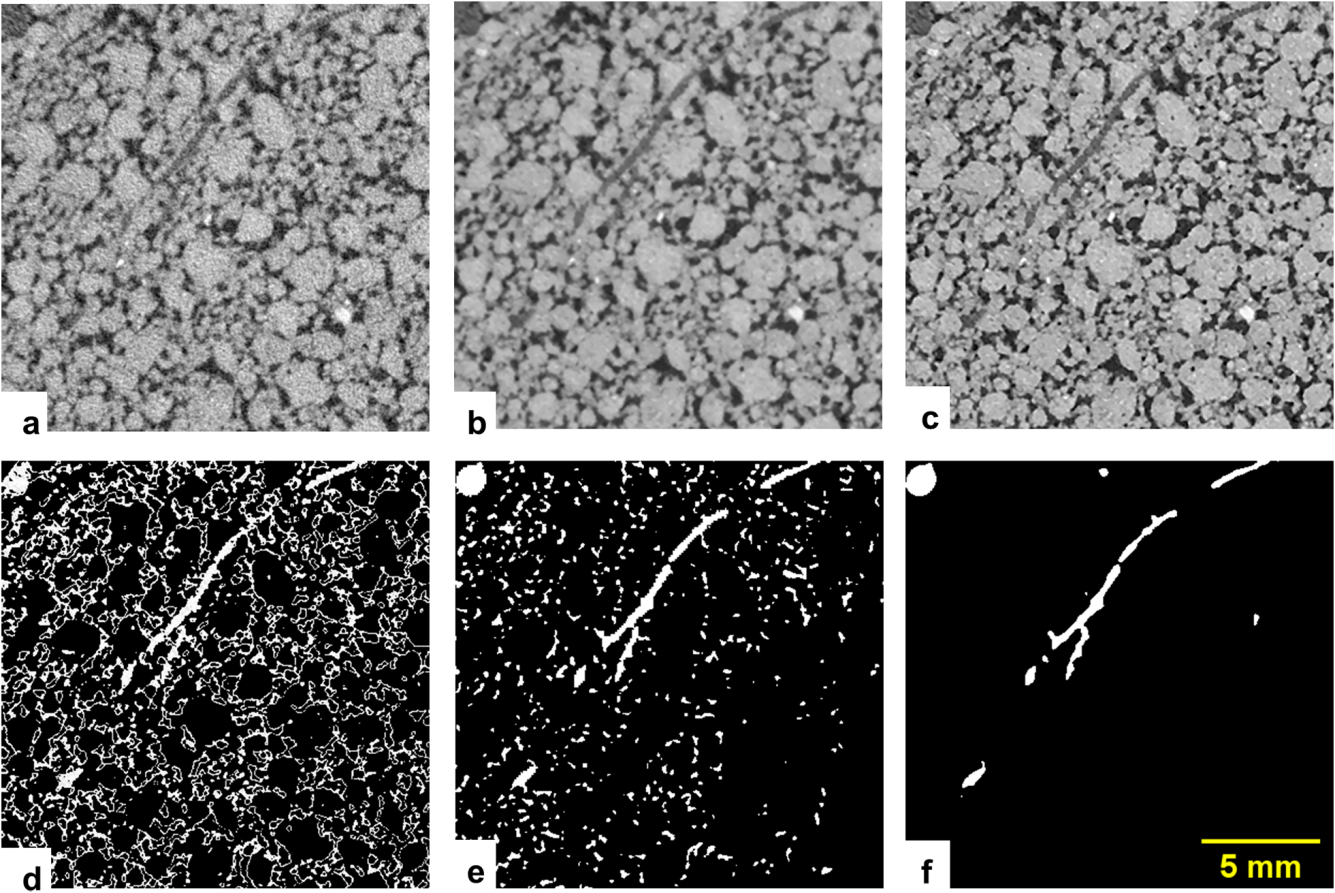
Results of the steps of Rootine v.2 for a subvolume of the worse case scenario. **a** The original grayscale image. **b** The obtained image after denoising with the 3D NLM filter. **c** The obtained image after performing edge enhancement of **b**. **d** Resulting image after background removal with ADT on **c**. **e** Results of the root segmentation applied on **d** before applying postprocessing steps. **f** Segmented roots after applying the postprocessing steps on **e**

Once the GV discontinuity of the stitched stack is corrected for, a filtering step is performed with a “3D Non-local Means (NLM)” filter [23]. This filtering step is performed with a plugin available in the ITK library [24]. This filter was chosen as it is fast [23] and can easily be incorporated in the workflow thanks to its standalone application. Note that in contrast to [6] (see Fig. S3 in [6]), we converted the images to 16-bit and added a constant GV offset of 50,000 to avoid the change of contrast inherent to the use of this filter. The change in contrast is an outcome of the Rician noise model implemented in this filter [23] as it was originally implemented for Magnetic Resonance Imaging. Avoiding the non-linear contrast enhancement for the low attenuation materials makes the results directly comparable to other softwares implementing a 3D NLM filter (e.g. Avizo™). The strength of the filtering is determined by the parameter “Contrast threshold” (${t}_{con}$) which needs to be given by the user as an input parameter. It is adjusted to the standard deviation of the image noise assessed by histogram analysis. Similarly to [6], the remaining parameters of the 3D NLM filter were set to default. The result of the 3D NLM filtering can be assessed by comparing the original grayscale data (Fig. 2a) and the filtered image (Fig. 2b) for a subvolume of the worse case scenario.

**Fig. 3:**
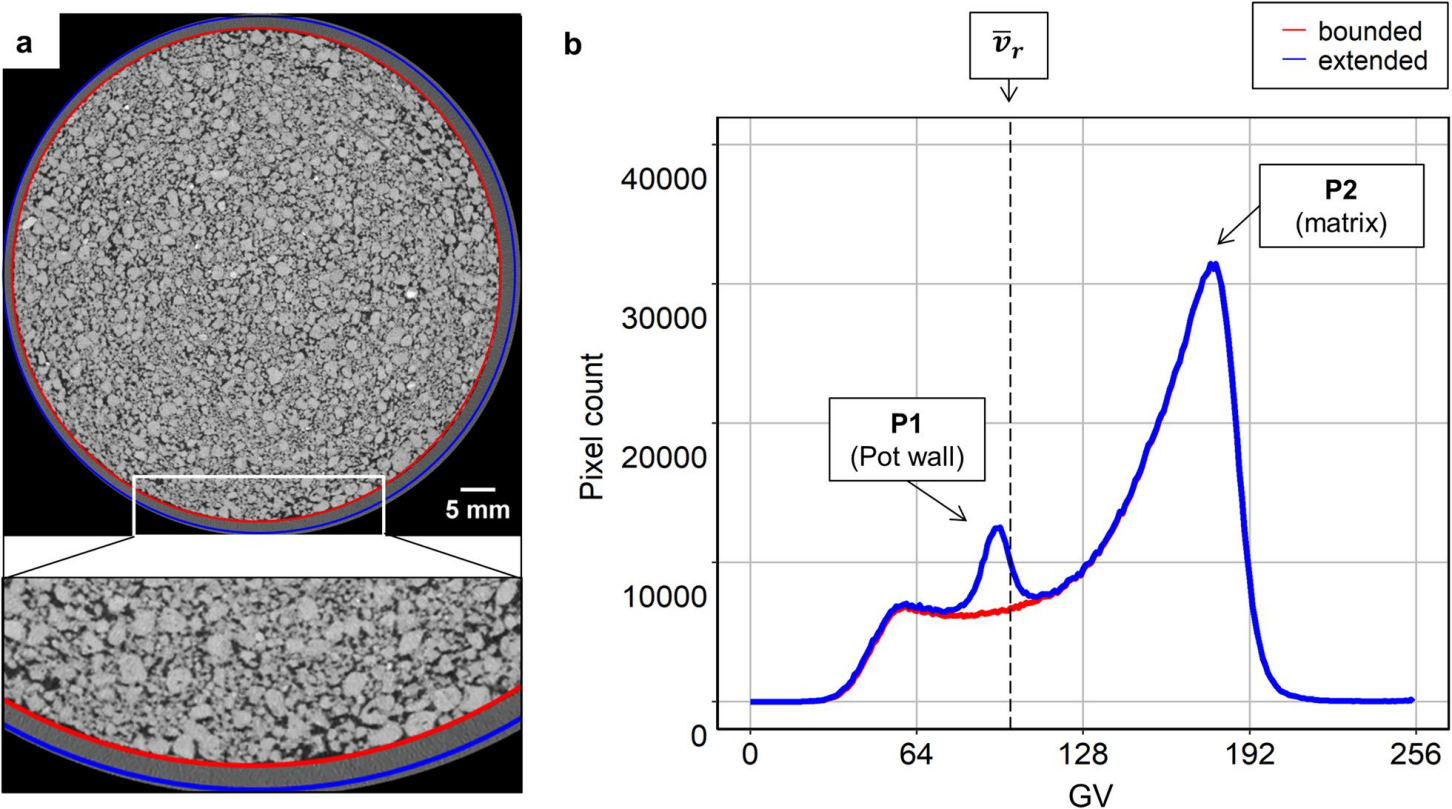
Mask creation and calculation of the average root GV based on characteristic peaks. **a** Depicts the drawing of a circular ROI bounded within the pot wall (red circle) on a 2D section of the worse case scenario. The bounded ROI serves the purpose of creating a mask. By extension of the bounded ROI by 50 pixels, an extended ROI is created (blue line). **b** Histogram of the bounded and extended ROI illustrated in **a** The extended ROI serves the purpose of creating a peak in the histogram which is used to calculate the average root GV

Rootine v.2 features a new pot wall detection step. This step serves two purposes. The first purpose is to create a mask which will exclude the pot wall from the data to segment and the second is to use the characteristic GV of the pot wall to generate a peak in the histogram. The generated peak is used later on for the calculation of the average root GV during a background removal step. In order to create a mask which excludes the pot wall, the coordinates of a circular region of interest (ROI) bounded within the pot wall limit need to be defined manually at three Z slices of the stack, i.e., at the first, the middle and the last slices. Those three sets of coordinates need to be given as input. The coordinates of the bounded ROI for all Z slices are then linearly interpolated from the given X–Y coordinates of the bounded ROI at the three Z slices. This allows creating a 3D mask, i.e., a mask whose boundaries in the X and Y dimensions will move as the stack is explored in the Z dimension. Creating a 3D mask is necessary to cope with pots being tilted during the X-ray CT scanning. Note that, at the resolution used in the benchmark datasets and considering the pot height, a certain tilt of the pots is always present. For tilted pots, a 2D mask would result either in masking the roots growing along the pot wall or in the inclusion of the pot wall in the data to segment. With the 3D mask, a logical “AND” operation is used on the filtered image in order to remove the pot wall from the data to segment. Once the bounded ROI is calculated for all Z slices, an extended ROI is created by simple extension of the bounded ROI by 50 voxels (Fig. 3a). The extended ROI serves the purpose of including the pot wall in the histogram analysis of the stack so that a characteristic peak is generated. After extracting the histogram of the extended ROI, a function searches for the maxima in the lowest part (i.e., from 0 to 128) and in the highest part (i.e., from 128 to 255) of the histogram. This function then retrieves the GV of those maxima, i.e., P1 and P2 which correspond to the average GV of the pot wall and of the soil matrix, respectively (Fig. 3b). Those two values are used further down in the workflow during the background removal step.

An edge enhancement step is then applied with the “Unsharp Mask” filter in ImageJ. “Unsharp Mask” filters enhance the local contrast between root edges, the surrounding soil matrix and pores [25]. The degree of edge enhancement is controlled by two input parameters. “Blur radius” is the standard deviation of the blur radius of the Gaussian filter kernel and “Mask weight” determines the strength of the filtering. The result of the edge enhancement step can be assessed by comparing the image filtered with 3D NLM (Fig. 2b) and the image after “Unsharp Mask” (Fig. 2c).

A new feature of the Rootine v.2 algorithm is to apply a background removal step. During the background removal, every voxel whose GV deviates too much from the average root GV (${\stackrel{-}{v}}_{r})$ is masked out. This operation is performed in a three steps procedure. During the first step, a calculation of the root average GV is carried out using the previously identified characteristic peaks of the pot wall and the soil matrix. Assuming that a shift of $\mathrm{P}1$ and $\mathrm{P}2$ would result in similar shift of ${\stackrel{-}{v}}_{r}$, ${\stackrel{-}{v}}_{r}$ can be calculated for every image of the dataset using Eq. (1),

1 $${\stackrel{-}{v}}_{r} ={f}_{r} . \left(\mathrm{P}2-\mathrm{P}1\right)+\mathrm{P}1$$

where ${\stackrel{-}{v}}_{r}$ is the root average GV, ${f}_{r}$ is the root GV factor which has to be determined a priori on a representative test image and P1 and P2 are the characteristic peaks of the pot wall and the soil matrix, respectively. The first step allows coping with differently contrasted images in the dataset. Differences in image contrast are due to the percentile stretching method used during the 8-bit conversion when reconstructing the tomograms. Once ${\stackrel{-}{v}}_{r}$ is calculated, a second step named “Absolute Difference Transform” (ADT) is applied. The rationale of this step is to brighten the GV of the roots and darken all background voxels including pores and soil matrix, as both materials have a GV different than ${\stackrel{-}{v}}_{r}$. For every voxel, this is done by computing Eq. (2),

2 $${v}_{ADT}=255-\left|{v}_{f}\right. -\left.{\stackrel{-}{v}}_{r} \right|$$

where ${v}_{ADT}$ is the voxel GV after the ADT and ${v}_{f}$ is the voxel GV prior ADT. The background is then masked out by thresholding the image with a threshold value (${t}_{ADT}$) calculated with Eq. (3),

3 $${t}_{ADT}=255- \frac{{R}_{r}}{2}$$

where ${R}_{r}$ is the root GV range centered around ${\stackrel{-}{v}}_{r}$. ${R}_{r}$ has to be determined a priori on a representative test image and has to be given as a input parameter. The image obtained after background removal serves as the input image for the subsequent root segmentation step. This three-step procedure replaces the simple pore masking step in Rootine v.1. The result of the background removal step can be assessed by comparing the image after edge enhancement (Fig. 2c) and the image after background removal (Fig. 2d).

#### Root segmentation

A specificity of the Rootine algorithms is to segment roots by exploiting one of their inherent characteristics, i.e., their cylindrical shape. To do so, the “Tubeness filter” available in ImageJ is used. In brief, the tubeness filter performs a smoothing of the image and produces an image in which the GV are directly related to how similar an object is to a cylinder. Generally, a scaled approach is adopted, i.e., the same image is filtered with Gaussian filters of different strength determined by their *σ* values, segmented and then combined. For low *σ* values, the roots of small diameters will evoke high GV after tubeness filtering whereas the bigger roots will either appear hollow or display low GV after tubeness filtering. Increasing the *σ* values of the tubeness filter then results in the opposite effect, i.e., the roots of greater diameter appear brighter whereas the roots of smaller diameter will vanish. The obtained series of images are then segmented using the “3D Hysteresis thresholding” method available in the 3D ImageJ Suite [26] and then combined to reconstitute the full root system. Hysteresis thresholding is a segmentation method requiring two thresholds. With an upper threshold ($${t}_{hys}^{high})$$, seed regions definitively belonging to roots are determined. The upper threshold is less relevant for segmentation accuracy and can be set a priori. From the upper threshold, a region growing process connects all voxels brighter than a lower threshold ($${t}_{hys}^{low}$$). This region growing process improves edge continuity in gradient images [27] and the class assignment of partial volume voxels [28], thereby reducing the presence of false positives.

**Fig. 4:**
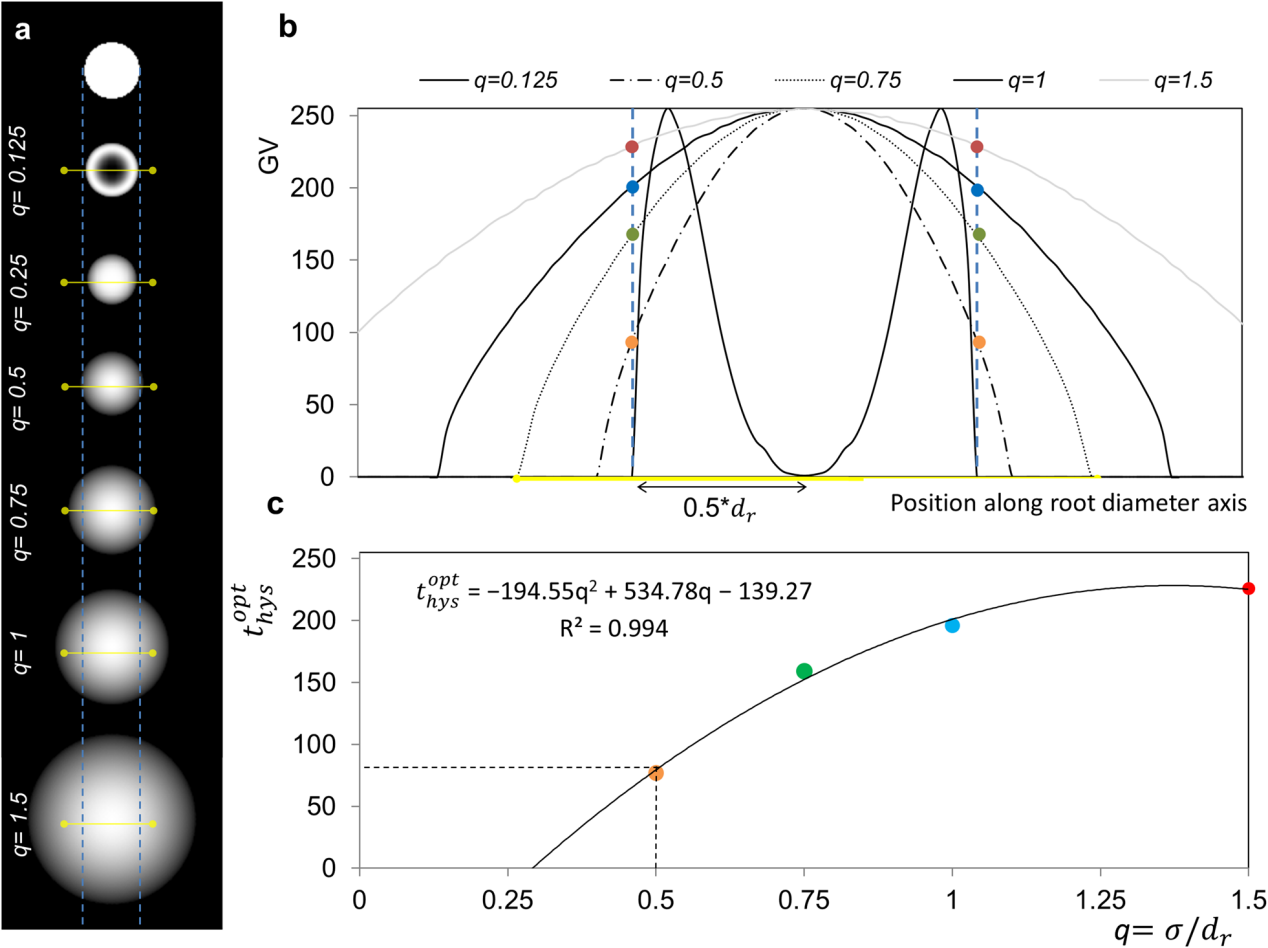
Estimation of σ values of the tubeness filters and the optimal lower thresholds of hysteresis thresholding. **a** Results of the tubeness filter on a hypothetical root of a diameter $${d}_{r}$$ obtained for different $$q$$ values. The dashed blue lines show the original root outline whereas the solid yellow lines show the position of the transects used to plot the GV along the root diameter axis. **b** Plot of GV along the root diameter axis for some of the $$q$$ values shown in **a**. The colored dots at the intersection between the root outline and the GV parabola correspond to $${t}_{hys}^{opt}$$ for a given $$q$$ value. **c** Line of best fit imposed on the couple of points $$q$$ and $${t}_{hys}^{opt}$$. In this study, we calculated $${t}_{hys}^{opt}$$ corresponding to = $$q$$0.5 using the model regression. The calculated value is indicated by the dashed line (i.e., $${t}_{hys}^{opt}=79)$$

**Fig. 5:**
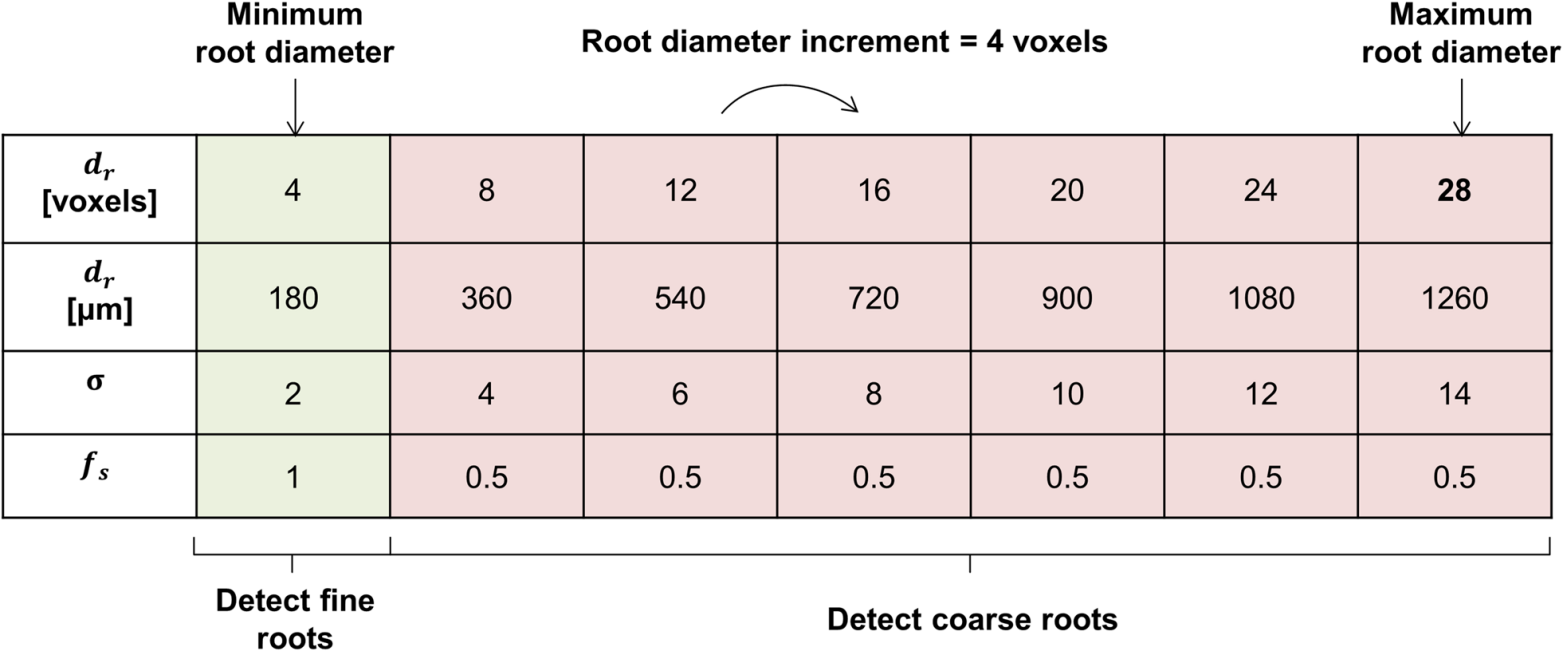
Approach for the detection of roots of increasing diameters at the original and the coarse resolutions

In this work, we introduce a new method to estimate the lower threshold applied during hysteresis thresholding. This estimation is based on the measurement of root diameters present in the image to segment. The link between the lower threshold applied during hysteresis thresholding and the root diameters has been made by analyzing carefully the results of the tubeness filter for increasing *σ* values applied on the same image of a hypothetical root (Fig. 4a). This was achieved with the following sequence of operations. First, a root having a diameter ${d}_{r}$ is created by drawing a white cylinder on a black background. Then, this root image is filtered with tubeness of increasing *σ* values. Note that the absolute value of the tubeness intensity depends on the gradient magnitude and hence the level of smoothing. Therefore, the contrast in the tubeness filter results were normalized, i.e., the highest GV after filtering is set to 255 during conversion from 32-bit to 8-bit. In order to generalize the obtained results, we introduce the normalized smoothing strength $\mathrm{q}$ which is equal to

4 $$q=\frac{\sigma }{{d}_{r}}$$

where *σ* is the smoothing strength of the tubeness filter and $${d}_{r}$$ is the root diameter. Both parameters are expressed in number of voxels. For each $$q$$ value, a GV transect along the root diameter axis is plotted (Fig. 4b). It is shown that, for low $$q$$ values, the filtered root appears hollow and the GV transect has symmetrical peaks on both sides of the root diametrical axis and a minimum exactly at the root diametrical axis. For $$q$$ values greater than 0.125, the transects have a concave parabolic shape with their symmetrical axis centered on the root diametrical axis. For a given $$q$$ value, the GV at the intersection of the parabola and the original root outline (i.e., the vertical blue dashed lines in Fig. 4a, b) corresponds to the optimal lower threshold ($${t}_{hys}^{opt}$$) to use during hysteresis thresholding in order to precisely capture the original root diameter. To formalize the calculation of $${t}_{hys}^{opt}$$, we retrieved $${t}_{hys}^{opt}$$ (i.e., the colored dots in Fig. 4b) for all $$\mathrm{q}$$ values and fitted a regression model (Fig. 4c) which describes best the relationship between those two parameters (i.e., highest possible R^2^ values). We then calculated $${t}_{hys}^{opt}$$ for *q* = 0.5 using the model regression (i.e., $${t}_{hys}^{opt}$$ = 79, see the dashed line on Fig. 4c). With the optimal lower threshold calculated and with a measurement of the diameter of the root to segment, the sigma value of the tubeness filter can be estimated (Eq. 5). It is important to note that, in case of image rescaling, the resolution factor ($${f}_{s})$$ needs to be accounted for in the measurement of $${d}_{r}$$. Thus, Eq. (4) is recast to:

5 $${\sigma }_{i}= q . {{d}_{r,i} . f}_{s}$$

where $${\sigma }_{i}$$ is the smoothing strength of the tubeness filter to use to properly segment a root of a certain diameter $${d}_{r,i}$$, *q* is the normalized smoothing strength and $${f}_{s}$$ is the resolution factor. For the segmentation of fine roots, the images were segmented at the original resolution (i.e., $${f}_{s}=1$$) whereas the bigger roots were segmented with an input image downscaled by a factor of 2 (i.e., $${f}_{s}=0.5$$) in order to reduce processing time without considerable loss of information. To determine values for $${d}_{r,i}$$ a priori, an increment approach was adopted to account for the continuous distribution of root diameters (Fig. 5). This approach requires three parameters, namely the minimum root diameter ($${d}_{r,min})$$, the root diameter increment ($${d}_{r,inc})$$ and the maximum root diameter ($${d}_{r,max})$$. All three parameters are expressed in units of number of voxels. The minimum and maximum root diameters were determined by measuring the diameter of the finest and biggest root in the image with the “Measure” tool available in ImageJ. The root diameter increment parameter refers to the increment at which roots of increasing diameters are detected. Here, a root diameter increment value equal to 4 voxels was set, which yields an incremented $$\upsigma$$ value of 1 at the coarse resolution according to Eq. (5). With these three parameters, an incremented calculation estimates the appropriate sigma values of tubeness for each resolution and scale considered and root diameters targeted. After filtering with tubeness and subsequent segmentation with hysteresis thresholding, the results were combined into one image with a logical “MAX” operation, i.e., a voxel is assigned to the root class if it is assigned to roots in at least one resolution or scale. This updated approach replaces the fixed scales and manually defined $${t}_{hys}^{low}$$ for each scale in Rootine v1. The result of the root segmentation step is in Fig. 2e.

#### Postprocessing

The postprocessing steps aim at removing artefacts which occurred in the course of segmentation. Such artefacts may include for instance segmented particulate organic matter or isolated pores whose GV are in the same range as the one of the roots. First, a 3D Median filter available in ImageJ is applied on the segmented images in order to smoothen the root surfaces. The degree of filtering is determined by the kernel size of the filter which needs to be given as an input parameter. On one hand, this filtering operation is favorable as it trims some over-segmentation voxels extending from the roots into the surroundings. On the other hand, this trimming also causes some fine root segments to be disconnected from the root system. After 3D Median filtering, the unconnected objects are removed using a connectivity criterion. This operation is performed with the “Keep Largest” function available in the “MorpholibJ” plugin library [29]. Prior to “Keep Largest”, an extra slice is added at the top of the stack to ensure the connectivity of all root segments from top to bottom. This is necessary when the seed from which all roots emerge is not part of the image. In the case of *Zea mays*, adding this step is essential as it allows keeping the brace and crown roots which do not directly emerge from the seed but always enter the ROI from the top.

A new feature of Rootine v.2 is to implement a “false negatives” recovery step. This step labels and evaluates every object unconnected to the root system and test whether it fulfills shape criteria which evoke the typical shape of roots. Those unconnected objects are either segmented clusters of pores and/or segmented particulate organic matter (i.e., false positives) or root segments which were disconnected due to the trimming effect of the previously applied 3D Median filter (i.e., false negatives). Here, we evaluate every unconnected object based on two criteria, i.e., its “Vesselness” and its size. To evaluate the vesselness, a simplified formulation of the vesselness function proposed by [19] was adopted and a “vesselness score” of individual objects was derived. This is based on the analysis of the length of the semi-axes of fitting ellipsoids to binary objects instead of evaluating the Hessian matrix (i.e., the 2nd derivative of GV) of each voxel. The semi-axes of the fitting ellipsoids are denoted as ${\lambda }_{1}$, ${\lambda }_{2}$ and ${\lambda }_{3}$. By convention and in order to make abstraction of the local orientation of the considered object in the 3D space, we pose ${\lambda }_{1}\le {\lambda }_{2} {\le \lambda }_{3}$. For every object, we then compute the following geometrical ratios:

6 $$Rb= \frac{{\lambda }_{1}}{\sqrt{{\lambda }_{2}*{\lambda }_{3}}}$$

7 $$Ra= \frac{{\lambda }_{2}}{{\lambda }_{3}}$$

The first ratio accounts for the deviation from a blob-like structure. For a blob-like object (i.e., ${\lambda }_{1}\approx {\lambda }_{2} {\approx \lambda }_{3}$), $Rb$ will attain high values whereas it will have low values for elongated objects (i.e., ${\lambda }_{1}\approx {\lambda }_{2} {\ll \lambda }_{3}$). The second ratio is essential for distinguishing between plate-like and cylinder-like structures. For a plate-like object (i.e., ${\lambda }_{1}\ll {\lambda }_{2} {\approx \lambda }_{3}$), $Ra$ will reach its maximum whereas it will be low for elongated objects. Based on the defined ratios, we evaluate how similar an object is to a cylinder by deriving its vesselness score $(\nu )$ according to Eq. (8).

8 $$\nu =\mathrm{exp}\left(-R{b}^{2}\right) . \mathrm{exp}(-R{a}^{2})$$

**Fig. 6:**
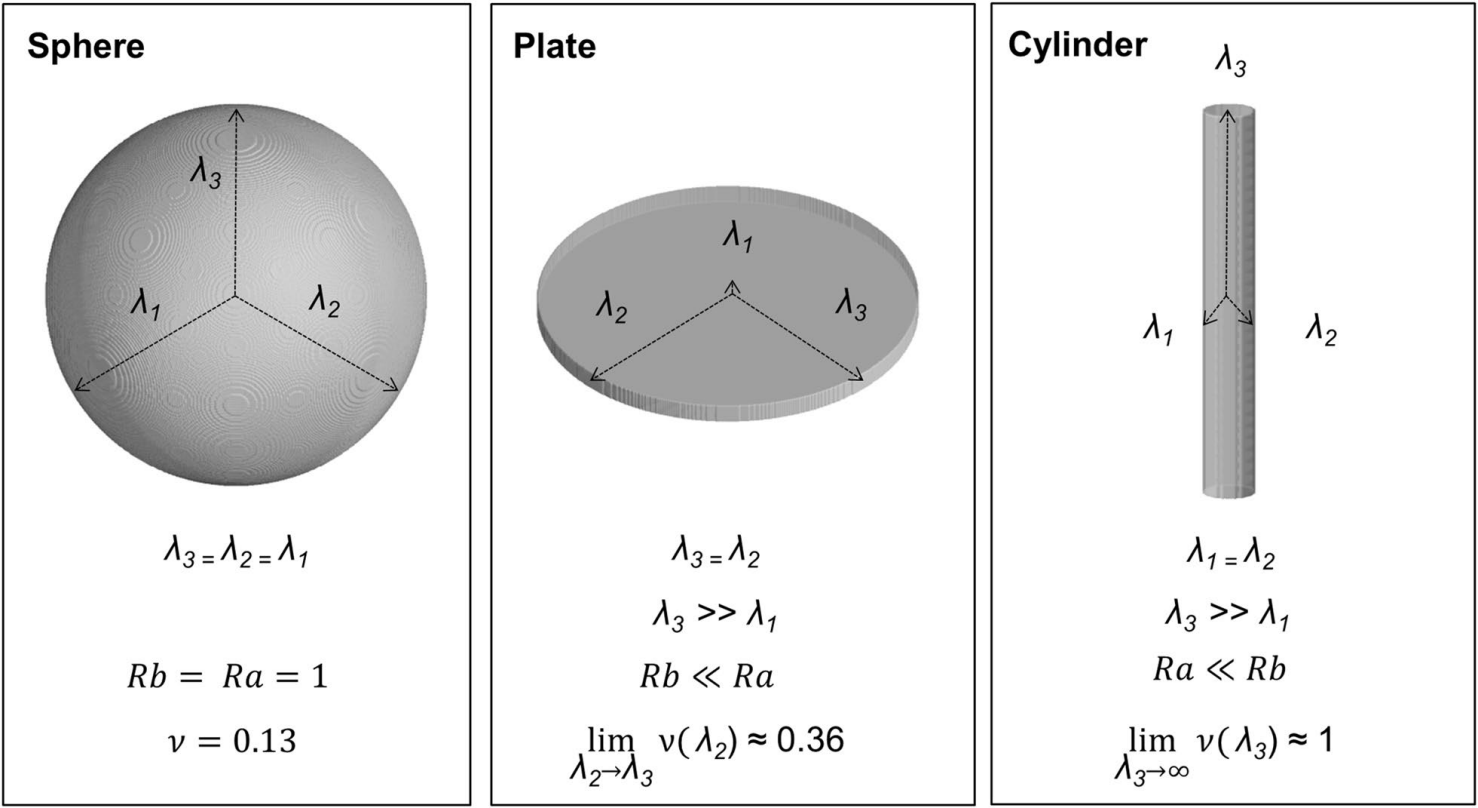
Vesselness score, *Rb* and *Ra* values for a sphere, a plate and a cylinder

**Fig. 7:**
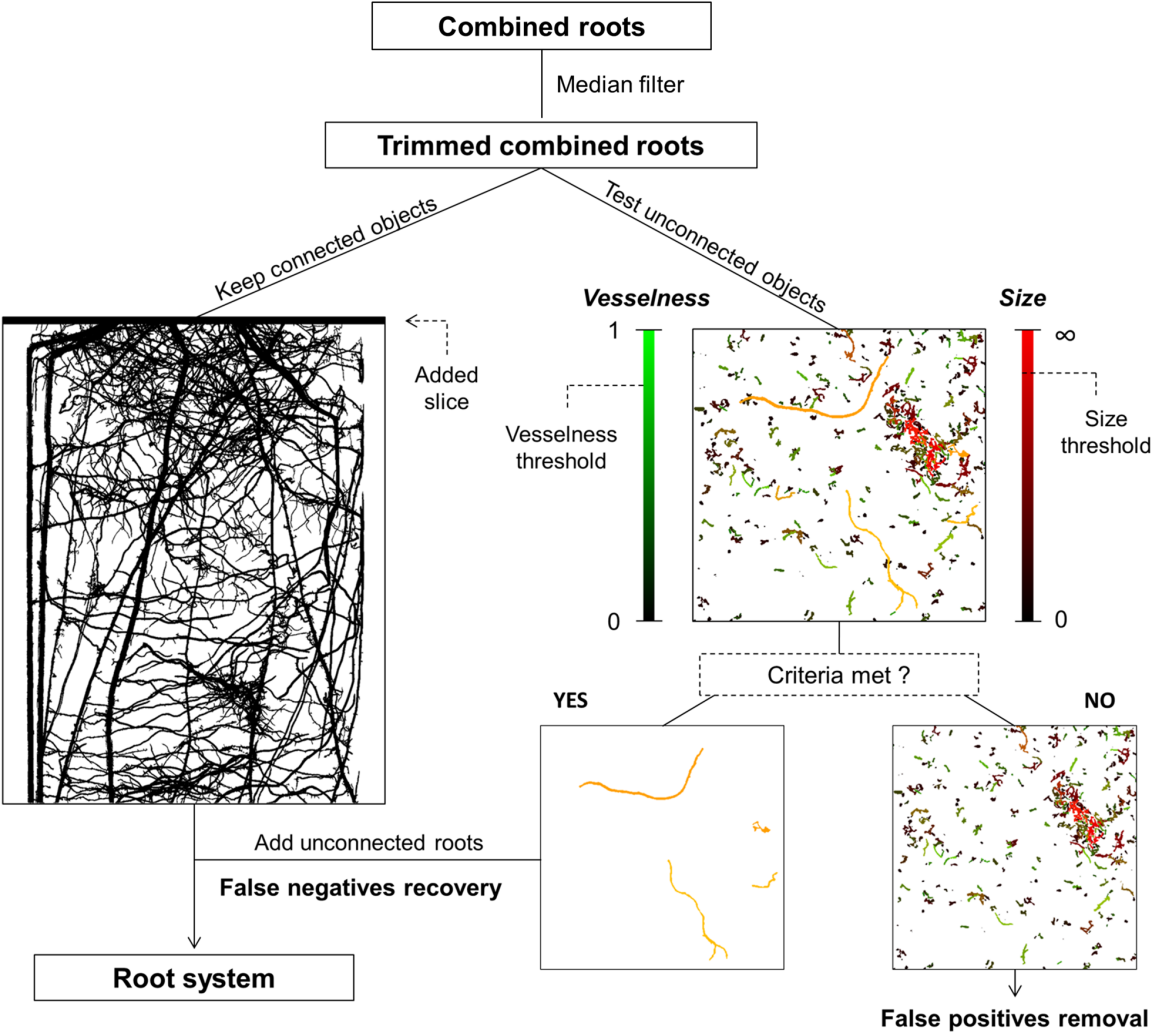
Illustration of the postprocessing steps implemented in Rootine v.2. First, a 3D Median filter is applied on the results of the root segmentation step. Then, all connected objects are kept by applying the “Keep Largest” function. In order to ensure full connectivity of the roots at the top of the stack, a slice is added at the top (left-hand side of the figure). The remaining unconnected objects are subjected to a test evaluating their shape, i.e., their “vesselness” and size. This is illustrated here by showing a Z-Projection of a 400 × 400 × 400 image from the best case scenario dataset (right-hand side of the figure). The green scale bar indicates the vesselness score whereas the red scale bar indicates the size score. The intensity of the yellow color depicts the combination of these two scores. If an object meets both the vesselness and size threshold, it is considered as a false negative and will subsequently be added to the connected root system. If not, it will be considered as a false positive and will be discarded

The vesselness score can have values ranging from 0.13 for a perfect sphere to ≈ 1 for an infinitely long cylinder. The relationship between the length of the semi-axes of the fitting ellipsoids, the calculated geometrical ratios and some properties of the vesselness score are illustrated in Fig. 6 for simple geometrical objects, i.e., a sphere, a plate and a cylinder. In addition to the vesselness criterion, a size criterion is used in order to exclude small objects which originate mostly from the noise level in the image and may by chance fulfill the vesselness criterion. The size criterion is given by a single value being equal to the greatest length of the semi-axes of the fitting ellipsoid, i.e., ${\lambda }_{3}$. In practice, evaluating the unconnected objects is performed in three steps. All steps rely on operations available in the “MorpholibJ” plugin library. Firstly, a label is assigned to every unconnected object via the “Connected Components Labeling" function. Secondly, for every unconnected object, the length of the semi-axes ${\lambda }_{1}$, ${\lambda }_{2}$ and ${\lambda }_{3}$ of the fitting ellipsoids are computed with the “Analyze Regions 3D” function. Thirdly, the vesselness score is calculated and assigned to each label using the “Assign Measure to Label” function. An object is then considered a false negative only if the following conditions are met:

$${\nu }_{i}>{t}_{v} \space \text { and  } {\lambda }_{3,i }> {t}_{s}$$

where ${\nu }_{i}$ and ${\lambda }_{3,i}$ are the vesselness and the size score of the object i and ${t}_{v}$ and ${t}_{s}$ are the vesselness and the size threshold, respectively. The vesselness and the size threshold are input parameters which need to be given and calibrated by the user. After discriminating between false positives and false negatives, the false negatives are added to the connected root system whereas the false positives are discarded. Figure 7 illustrates this new approach of the postprocessing scheme implemented in Rootine v.2. The effect of the post processing steps is shown in the difference between Fig. 2e, f.

#### Quantification and analysis

Following postprocessing, the images can be analyzed and quantified in terms of root length and diameter. The quantification of root length from X-ray CT data has to be preceded by a step of skeletonization which conducts a medial axis transform of the segmented root image. This results in an image where all roots are reduced to a 1 pixel wide object which makes the calculations of root length more reliable and faster. This is achieved by sequentially applying the “Skeletonize (2D/3D)” and “Analyze Skeleton (2D/3D)” methods available in the BoneJ plugin library [30]. The root recovery is assessed by plotting the root length calculated after skeletonization of the segmented root system and the root length analyzed with WinRHIZO (WR). By imposing a line of best fit to the relationship between both root lengths (CT and WR), the root recovery and the error consistency (i.e., the slope and the coefficient of determination of the line of best fit, respectively) can be evaluated. The quantification of the root diameter distribution is performed with the “Local Thickness” plugin available in BoneJ. This method assigns to every root voxel a value corresponding to the diameter of the largest sphere which locally fits into the root. The results of the local thickness images are intersected with the skeleton images with a logical “AND” operation. The resulting images are skeletonized root systems where each medial axis voxel contains the local root diameter information. This intersection is performed in order to avoid that big roots contribute to more voxels than smaller roots in the histogram. The histogram of the obtained images is then computed to retrieve the root length corresponding to every diameter class. Note that, even though roots were destructively sampled at two different layers for six replicates, the results are shown here by pooling all replicates and all layers together for each scenario. For both the root length and the root diameter distribution, the root length is normalized by dividing by the volume of the soil layer and the results are expressed in terms of RLD.

#### Summary of the workflow and its parameters

This section concludes the description of the workflow of Rootine v.2. Figure 1 and Table 1 provide an overview of the steps of the workflow and the tunable parameters involved to obtain a segmented root system from an input grayscale data acquired with X-ray CT. Table 1 also list the effect and the sensitivity of the parameters on the segmentation accuracy. Note that the effect and sensitivity of the parameters have been assessed visually thanks to the acquired user-experience during the calibration of the method for our specific dataset. The mention of the sensitivity of the parameters has the sole purpose of giving general advice to potential users during the calibration of Rootine v.2 for their specific dataset.

**Table 1:** Summary of the parameters, their values, their effects and their sensitivity on the segmentation accuracy

Step	Parameter	Value		Effect	Sensitivity on the segmentation accuracy
		Worse case	Best case		
Image filtering	Contrast threshold (${t}_{con}$)	60	60	Controls the degree of smoothening (i.e., noise removal) of the input image	Medium
Edge enhancement	Blur radius	1	0.9	Both parameters control the degree of sharpening of the image, i.e., increase the contrast at the boundaries between roots and pores and soil matrix	High
	Mask weight	0.7	0.8		
Background removal	Root gray value factor (${f}_{r}$*)*	0.10	0.18	Sets the average gray value of the roots	Very high
	Root gray value range (${R}_{r}$)	65	70	Controls the root gray value window around the average root gray value. If set too high, overestimation of roots into their surroundings will occur. If set too low, loss of roots will occur	Very high
Detect fine roots	Minimum root diameter (${d}_{r,min}$)	4	4	Controls the root recovery of the fine roots. If set too high, the fine roots are not detected. If set too low, over-segmentation may occur depending on the noise level of the image	High
Detect coarse roots	Maximum root diameter (${d}_{r,max}$)	28	28	Controls the accuracy of the root diameter outline of the biggest root. If set too high, the diameter of the biggest root is overestimated and computational time is wasted. If set too low, the diameter of the biggest root is underestimated	Medium
False positives removal	Kernel size of median filter	3	2	Controls the degree of smoothening of the roots and trimming of over-segmented voxels. If set too high, root loss occurs whereas low values result in the presence of false positives	High
False negatives recovery	Size threshold (${t}_{s}$)	25	25	Both parameters control the quality of the false negatives added in the root system. If set too low, many false positives are considered negatives, If set too high, root loss occurs	High
	Vesselness threshold (${t}_{v}$)	0.85	0.9		High

## Results

### Root recovery and root diameter evaluation

**Fig. 8:**
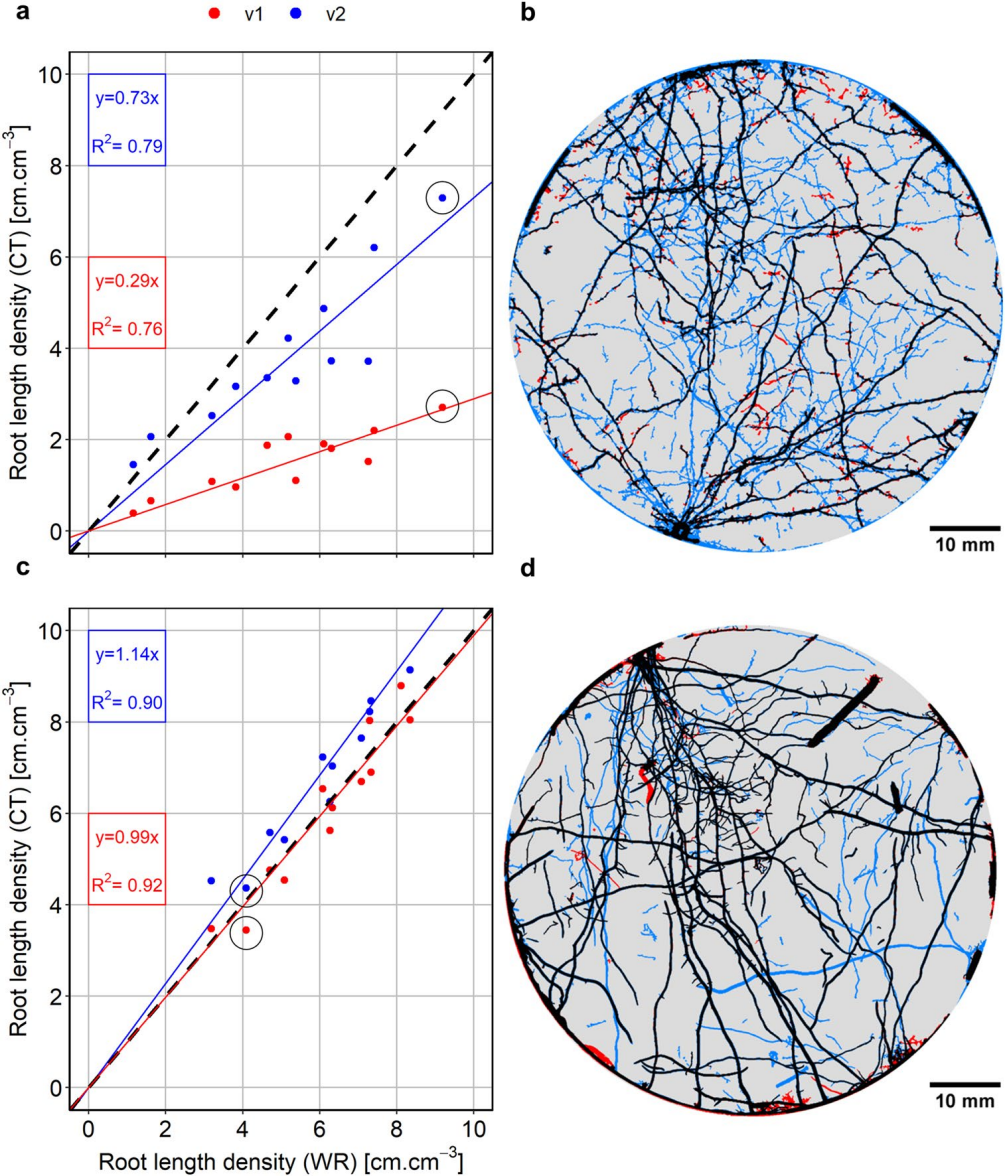
Root recovery of Rootine v.2 for the worse and best case scenario. **a**, **c** Comparison with the former Rootine v.1 and the RLD determined with destructive sampling and scanning of washed-off roots (WinRHIZO) for the worse case and the best case scenario, respectively. The dashed line indicates the 1:1 line. **b**, **d** Visual comparison of the segmented root systems obtained with Rootine v.1 and Rootine v.2 for the corresponding sample circled in black on **a** and **c** for the worse case and the best case scenario, respectively. Roots detected by both algorithms are depicted in black, the ones only detected by Rootine v.2 are shown in blue, whereas roots only detected by Rootine v.1 are shown in red

**Fig. 9:**
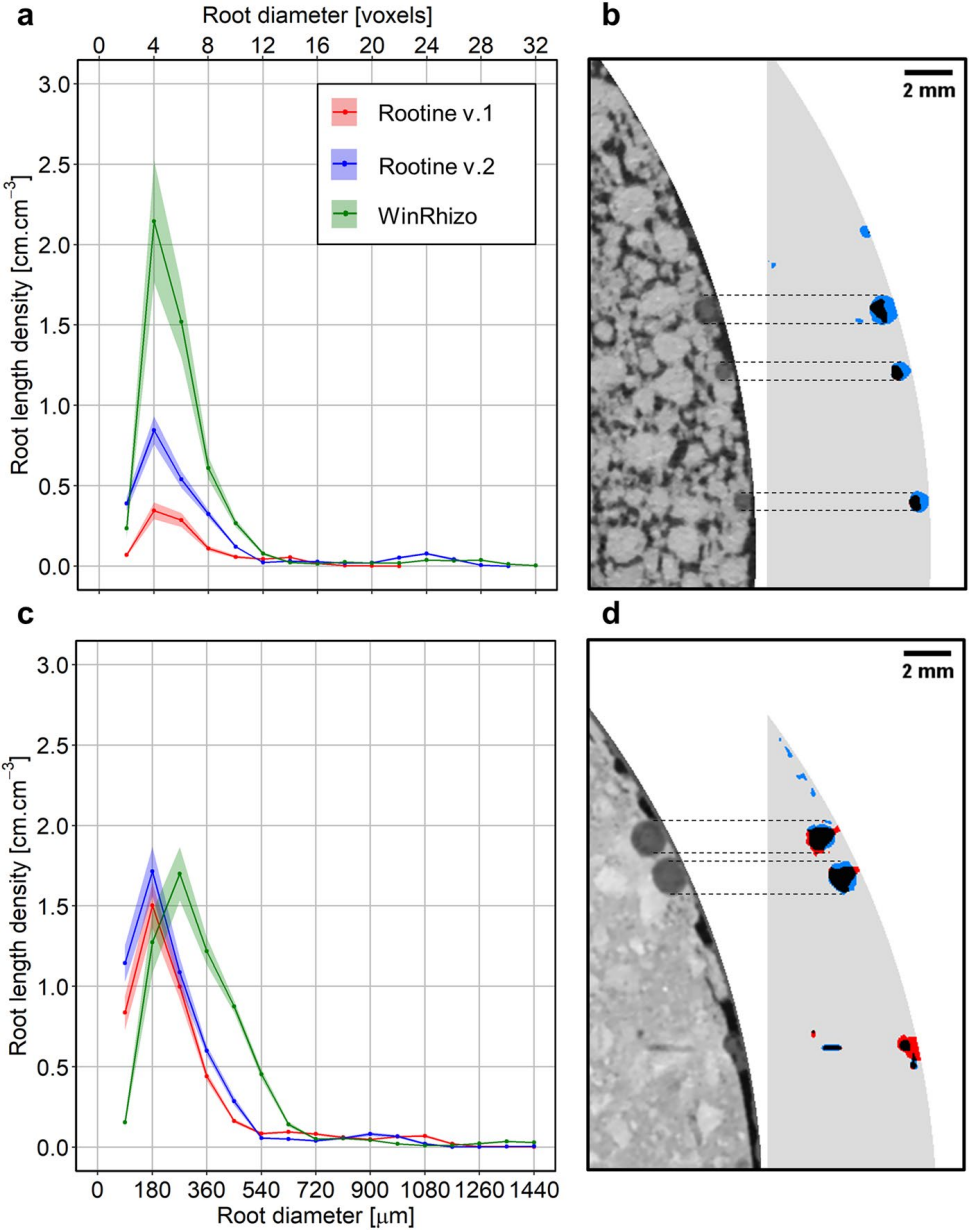
Root diameter distribution and root outline accuracy for the worse and best case scenario. **a**, **c** RLD distribution as a function of root diameter for Rootine v.1 and v.2 and the destructive sampling data obtained by scanning washed-off roots (WinRHIZO) for the worse case and the best case scenario, respectively. The semitransparent ribbon denotes the standard error of the measurements (n = 12). **b**, **d** Visual comparison of the segmented root diameter outlines for both Rootine v.1 and Rootine v.2 supported by the original X-ray CT grayscale data for the worse case and the best case scenario, respectively. Roots detected by both algorithms are depicted in black, the ones only detected by Rootine v.2 are shown in blue, whereas roots only detected by Rootine v.1 are shown in red. Dashed horizontal black lines highlight the fact that Rootine v.2 better captures root diameter in comparison with Rootine v.1

**Fig. 10:**
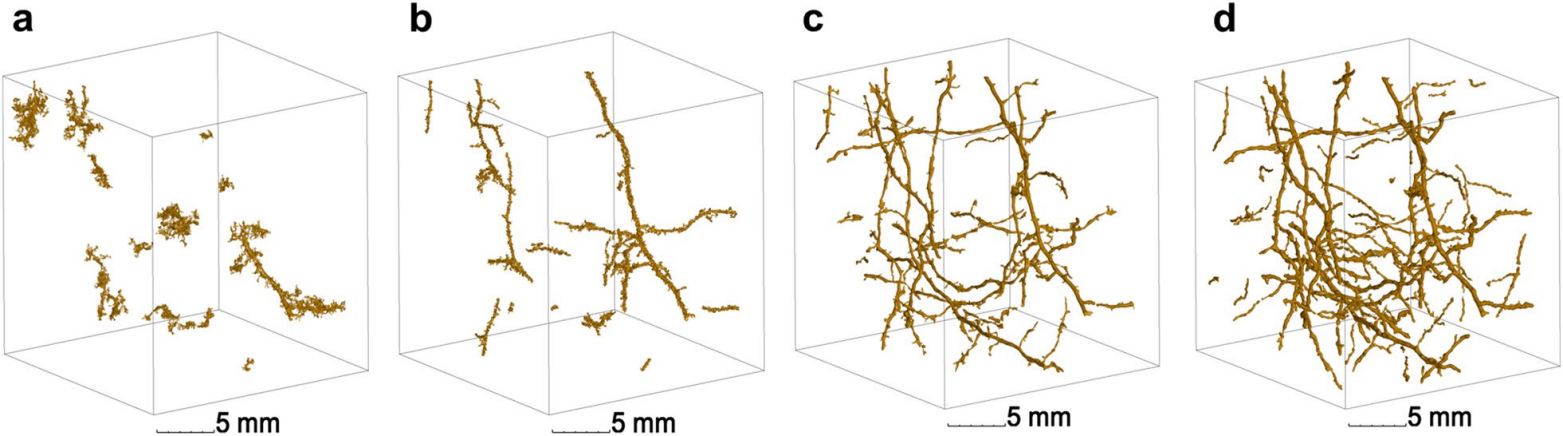
Results of Rootine v.2 and v.1, Root1 and Region growing for a subvolume from the worse case scenario. **a** Results obtained with Region growing. **b** Results obtained with Root1. **c** Results obtained with Rootine v.1. **d** Results obtained with Rootine v.2

In the worse case scenario, Rootine v.2 outperformed its preceding version by an increase of the root recovery up to 73% of the total root length, against 29% for Rootine v.1 (Fig. 8a). The coefficient of determination is roughly equal for both algorithms (i.e., R^2^ = 0.76 and 0.79 for Rootine v.1 and v.2, respectively). A 2D Maximum Z-Projection of a selected sample (circled in black in Fig. 8a) shows that the over-segmentation is low (Fig. 8b). Detected roots appear relatively smooth and there are barely any root voxels extending into their surroundings. In the 2D Maximum Z-Projection, some root segments are disconnected from the root system. Those segments are the ones added by the false negatives recovery step during postprocessing. Figure 8b also shows that the gain in root length with Rootine v.2 is mainly contributed by additional fine roots (operationally defined as roots having a diameter ≤ 180 μm). The increased fine root recovery is also reflected in the root diameter distribution (Fig. 9a). On top of a higher root recovery of the fine roots, Rootine v.2 also better captured the root diameter of the big roots (operationally defined as roots having a diameter ≥ 900 μm) as compared to its preceding version. This can be seen on Fig. 9a where Rootine v.2 agrees better with WR data for the diameter classes larger than 900 μm as compared to Rootine v.1. The second peak (corresponding to the primary roots) only underestimates WR values by 4 voxels for Rootine v.2, whereas this second peak is completely absent for Rootine v.1. The better agreement of the root diameter distribution with Rootine v.2 can also be assessed visually by superimposing the segmented images of both algorithms and by directly comparing them with the grayscale X-ray CT data (Fig. 9b). The visual comparison of the results obtained with the region growing method, the Root1 algorithm, Rootine v.1 and Rootine v.2 for a subvolume of the worse case scenario shows that Rootine v.2 outperformed other root segmentation state of the art methods as well (Fig. 10).

In the best case scenario, the root recovery amounts to 114% of the total root length against 99% for Rootine v.2 and Rootine v.1, respectively (Fig. 8c). Again, the coefficient of determination is roughly equal for both versions (i.e., R^2^ = 0.92 and 0.90 for Rootine v.1 and v.2, respectively). Similarly to the worse case scenario, a 2D Maximum Z-Projection of the segmented roots of a selected sample (Fig. 8b) offers a visual comparison of the segmentation results of both versions. Here again, the over-segmentation is low as roots appear smooth and devoid of any over-segmented voxels at their boundaries. The increase in root recovery is also mostly contributed by the addition of fine roots. The agreement of the root diameter distribution is equally good for both versions (Fig. 9c).

## Discussions

### Segmentation accuracy

In the worse case scenario, most of the roots that were missed by Rootine v.1 and v.2 belonged to the category of the fine roots. This was expected considering that one of the main challenges of the benchmark dataset is a low image resolution as compared to the small diameter of the fine maize roots. Indeed, the analysis of the cumulative frequency of root diameter of the WR data revealed that the fine roots comprise roughly 45% of the total root length. Fine roots pose a tremendous challenge due to the presence of partial volume voxels at the boundary of the roots and their surroundings. This challenge is even bigger when the contrast between the roots and their surroundings is low, which was true for the worse case scenario. We attempted to capture more fine roots at the original resolution by reducing the minimum root diameter to two voxels (i.e., resulting in a smoothing strength of *σ* = 1). However, this resulted in too much over-segmentation. Still, Rootine v.2 was able to capture twice as much of the fine roots as compared to Rootine v.1 in the worse case scenario. This can be attributed to the background removal step during preprocessing and to the false negatives recovery step during postprocessing. The background removal prevented the presence of false positives even when segmenting the images at the original resolution. In contrast, the soil heterogeneity and the low signal-to-noise ratio of the worse case scenario forced [6] to downscale the images by a factor of 2 prior root segmentation. The authors argued that segmenting the images at the original resolution resulted in too much over-segmentation. By applying an adequate background removal operation, Rootine v.2 was able to avoid over-segmentation while improving the recovery of fine roots considerably. On top of an adequate background removal, the ADT step increases the GV intensities of the roots prior tubeness filtering which is favorable for their subsequent detection. The false negatives recovery step also contributed to a fair amount of the root recovery. By computing the RLD before and after this step, this contribution can be evaluated. On average, it amounted to 25.5% (± 1.9% standard error, n = 12) of the total RLD. So the false negatives recovery step explained more than half of the gain in root recovery between Rootine v.1 and v.2.

In the best case scenario, the RLD inferred from X-ray CT was higher than the one measured with WR. This is an indication of the presence of false positives and/or over-segmentation at the boundaries of root voxels. We rule out the latter since the visual inspection of the images showed virtually no root voxels extending into their surroundings. The overestimation of root length with Rootine v.2 could be due to the false negatives recovery step during which some actual false “positives” were considered as being false “negatives” and added back to the root system. For the best case scenario, the contribution of this step to the total RLD was lower than for the worse case scenario and amounted to 12.5% (± 0.8% standard error, n = 12). The overestimation of the root recovery could also be due to the uncertainties associated with the root washing procedure and further analysis with WR. The Z-Projection shown in Fig. 8d is one of the samples whose X-ray CT RLD data point exceeded the RLD data measured with WR (i.e., the sample circled in black above the 1:1 line in Fig. 8c). When taking a closer look at Fig. 8d, it is obvious that the increase in root recovery is partly due to the addition of real roots. During root washing, a soil sample is placed on a sieve, the soil is then washed off with water and the roots remaining on the sieve (here having a 1 mm mesh size) are picked with a tweezer and stored in ethanol prior to analysis. Some fine roots can easily go unnoticed on the sieve due to their size. The fact that the overestimation of WR data by Rootine v.2 occurs specifically for the fine roots is an indication supporting this argument. On top of the root washing, errors in WR data might be induced by an uneven distribution of the roots on the tray during scanning. An uneven distribution of the roots might cause two fine roots located very close to each other to be detected as one root with a larger diameter instead. During the analysis with the WinRHIZO software, a noise threshold value has to be set to exclude small dirt particles from the root length calculation. A high noise threshold value leads to smooth root surfaces but also results in the loss of fine roots. An improper setting of this parameter may then also induce errors. We rule out the effect of storing roots in ethanol on the WR results as this procedure has proved to be valid to conserve root samples without considerable influence on the measurements of root length (i.e., < 1% of underestimation) and diameter (i.e., 5% of underestimation) [31]. Both the potential loss of roots during washing and the underestimated detection of fine roots by WinRHIZO could explain the overestimation of the root recovery in the best case scenario. Note that if the RLD data characterized with WR is underestimated, it is likely that the root recovery in the worse case scenario is overestimated.

The segmentation accuracy was evaluated based on quantitative aspects such as the root recovery and the comparison of root diameter distribution. Additionally, the segmentation accuracy was also evaluated visually based on qualitative aspects, i.e., how accurately the root diameter outlines were segmented. With Rootine v.1, the primary roots often showed irregular shapes. With Rootine v.2, primary roots were segmented with a higher accuracy and showed a prominent circular shape when viewed in a 2D X–Y cross section. This can be seen in Fig. 9b, d. This increase in accuracy can most likely be ascribed to the fact that more scales were considered during the tubeness filtering at the coarse resolution with Rootine v.2. However, this difference in capturing the root diameter outlines was not big enough to be reflected in the root diameter distribution in the best case scenario. By visually comparing the results of Rootine v.1 and v.2, it was also noticeable that the latest version showed less false positives and less segmentation artefacts. Such segmentation artefacts include for instance the over-segmentation of roots growing along the plastic wall of the pot. Rootine v.1 did not feature a pot wall detection and removal step. Since the root average GV is close to the one of the pot wall (see the corresponding GV of ${\stackrel{-}{v}}_{r}$ and P1 in Fig. 3b), it was often observed on the results of Rootine v.1 that segmented root were extending into the plastic wall. This was particularly true in the best case scenario. With Rootine v.2, the pot removal step prevented that from happening.

### Numbers of tunable parameters

One of the objectives of this work was to develop an algorithm in which the numbers of tunable parameters is reduced. Note that we consider as “tunable” the parameters which require adjustments and calibration when applied to other datasets (i.e., experiments with different plants, scan settings and/or soil heterogeneity).

In Rootine v.1, we identified twelve tunable parameters in total, namely five for the preprocessing, six for the root segmentation and one for the postprocessing. For the preprocessing, one parameter was used for filtering the original grayscale image (i.e., the contrast threshold of the 3D NLM filter), two for edge enhancement (i.e., the blur radius and the mask weight of the Unsharp mask filter) and two for masking the pores by single thresholding (pores were masked with different thresholds at the original and at the coarse resolution). For the root segmentation step at the original resolution, one *σ* value and the corresponding $${t}_{hys}^{low}$$ for hysteresis thresholding method were used. For the root segmentation step at the coarse resolution, three *σ* values were used. The obtained results of the tubeness filtering were then merged and one $${t}_{hys}^{low}$$ for the hysteresis thresholding method was used to segment the results of the coarse root detection (this amounts to four parameters). The upper threshold for hysteresis thresholding ($${t}_{hys}^{high})$$ was kept constant and high enough for every tubeness filtering scale and is thus considered non-tunable. For the postprocessing, Rootine v.1 relied on one parameter, i.e., the kernel size of the 3D Median filter.

In Rootine v.2, the pore masking thresholds were replaced by the root GV factor ($${f}_{r})$$ and the root GV range ($${R}_{r})$$. The parameters of the 3D NLM filter and the Unsharp mask filtering are also used in the new version. With the introduction of the automatic calculation of the *σ* values and $${t}_{hys}^{opt}$$ and keeping $${t}_{hys}^{high}$$ high enough and constant for every tubeness filtering scale, the number of parameters required for the root segmentation was reduced to two (i.e., $${d}_{r,min}$$, $${d}_{r,max}$$). As they have been set once for the new root segmentation approach, we consider the parameters *q*, $${f}_{s}$$ and $${d}_{r,inc}$$ as “quasi-fixed” and therefore non-tunable. For the postprocessing, a smoothening step of the root outline was performed with the “3D Median” filtering step requiring one parameter (i.e., the kernel size). The false negatives recovery step added two parameters, i.e., the vesselness $$\left({t}_{v}\right)$$ and the size $$({t}_{s})$$ thresholds. In total, the number of tunable parameters in Rootine v.2 was reduced to ten, i.e., five for the preprocessing of the image, two for the root segmentation and three for the postprocessing. It is worth noting that more parameters are required to use the full functionalities of Rootine v.2, i.e., the coordinates of the ROI mask. Those coordinates need to be directly evaluated on the image and are not considered to influence the segmentation results if appropriate values are given. The comparison between the tunable parameters used in Rootine v.1 and Rootine v.2 for every image processing step is shown in Table 2.

**Table 2:**
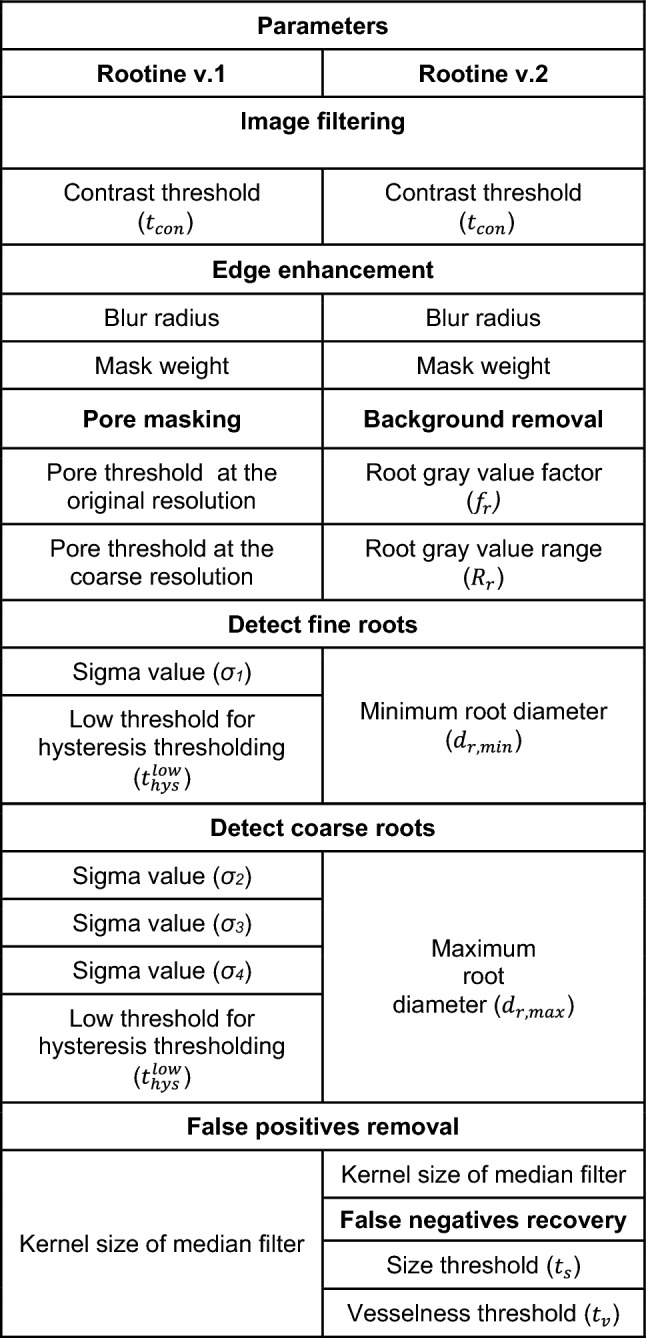
Tunable parameters used in Rootine v.1 and v.2

### Runtime and overall usability of Rootine v.1 and v.2

Besides the segmentation accuracy and the number of parameters, the assessment of the performance of a segmentation algorithm also has to take into account the time it takes to process the images. This is important for application in high-throughput root phenotyping based on X-ray CT data analysis. To process a stack having a dimension of 1750 × 1750 × 3000 voxels (in X, Y and Z dimension, size ≈ 8.6 GB), it took Rootine v.1 3.8 h to complete the preprocessing, the segmentation and the postprocessing steps. In comparison, Rootine v.2 took 6.8 h to complete the same steps. The second version is 1.8 times slower than the first version for the analyzed image size. This is mainly due to the consideration of more scales during tubeness filtering at the coarse resolution. For both algorithms, the evaluation of the runtime was performed on a workstation having 64 Intel^®^ Xeon^®^ Gold 6142 cores running at 2.60 GHz each. To this date, filtering with the tubeness filter represents the bottleneck of the workflow. This is related to the fact that the tubeness filter is only implemented in a single threaded fashion in the ImageJ software. There should be no fundamental constraint that would restrict its parallelization and Rootine (regardless of the versions) would benefit a lot from it. Note that there exists a multithreaded implementation of the tubeness plugin which was developed in the context of ImageJ Ops [32]. We have however not tested it. It is worth noting that both algorithms can be run in a user interaction-free mode (i.e., from the command line) once the parameters are adjusted. This provides an advantage and, in our opinion, reduces the necessity of having a fast algorithm as the macro can run in the background and/or overnight. When it comes to root system architecture studies, a longer runtime can be well accepted as long as the root recovery is substantially increased. Despite the longer time required to segment the images, we are confident that the increase of the overall usability of Rootine v.2 can save the user some time for the adjustments of the parameters. Indeed, Rootine v.2 features input parameters which are linked to root physiological properties such as their GV and their diameter. These tunable parameters are easy to adjust as they can be directly assessed visually on a test image.

## Conclusion

Rootine v.2 has been developed for improved root segmentation accuracy in X-ray CT data. It exploits intrinsic properties of root systems such as the connectivity of root branches and the cylindrical shape of roots to distinguish roots from the background. It was demonstrated that Rootine v.2 outperforms its precursor version in terms of root recovery as well as other state-of-the-art root segmentation methods. The gain in root recovery could be mainly ascribed to the absolute difference transform of the grayscale data prior to shape detection with a series of tubeness filters and to a false negatives recovery step. The other major advancements of Rootine v.2 are (i) a pot wall detection and removal step, (ii) a calculation of the root average gray value based on a histogram analysis and (iii) an automatic calculation of thresholds for hysteresis thresholding of the tubeness image. Moreover, the analysis of the root diameter distribution is readily integrated in the new version. The total number of tunable parameters for the entire workflow was reduced from twelve to ten. Rootine v.2, in comparison to Rootine v.1, functions less in a “black box” fashion as its parameters can be more easily interpreted and are easier to adjust. The proposed method has the potential of improving high-throughput root phenotyping procedures based on X-ray CT data analysis. Similarly to its preceding version, Rootine v.2 is a macro for the image processing software ImageJ and is made freely available to the public.

## Data Availability

The entire datasets used in this study are available from the corresponding author on reasonable request. The Rootine v.2 workflow and the CT images (approx. 6.5 GB each) of one pot of the worse case scenario are provided through the link: http://www.ufz.de/record/dmp/archive/10553/de/.

## Abbreviations

ADT: Absolute difference transform
CT: Computed tomography
GV: Gray value
NLM: Non-local means
ROI: Region of interest
RLD: Root length density
WR: WinRHIZO
